# Exploring the Peanut Viromes Across 15 Cultivars in Korea

**DOI:** 10.3390/ijms27020890

**Published:** 2026-01-15

**Authors:** Sang-Min Kim, Ki Wook Kwon, Yeonhwa Jo, Hoseong Choi, Jisoo Park, Jin-Sung Hong, Bong Choon Lee, Won Kyong Cho

**Affiliations:** 1 Crop Foundation Division, National Institute of Crop Science, Rural Development Administration, Wanju 55365, Republic of Korea; kimsangmin@korea.kr; 2 Sangju Persimmon Research Institute, Gyeongsangbuk-do Agricultural Research & Extension Services, Daegu 41404, Republic of Korea; kkm31501@korea.kr; 3 Department of Plant Protection and Quarantine, Jeonbuk National University, Jeonju 54896, Republic of Korea; yeonhwajo@gmail.com; 4 Biocube System, Inc., Suwon 16648, Republic of Korea; bioplanths@gmail.com; 5 Agriculture and Life Sciences Research Institute, Kangwon National University, Chuncheon 24341, Republic of Korea; pjsbob@gmail.com; 6 Interdisciplinary Program in Smart Agriculture, Kangwon National University, Chuncheon 24341, Republic of Korea; jinsunghong@kangwon.ac.kr; 7 Division of Crop Protection, National Institute of Agricultural Science, Rural Development Administration, Wanju 55365, Republic of Korea

**Keywords:** peanut cultivars, RNA sequencing, viral abundance, complete genomes, phylogenetic analysis, single nucleotide polymorphisms (SNPs), virome analysis, Korea

## Abstract

This study explores the virome of fifteen peanut cultivars in Korea. Through RNA sequencing, 305 viral contigs associated with cucumber mosaic virus (CMV), peanut mottle virus (PeMoV), bean common mosaic virus (BCMV), and brassica yellows virus (BrYV) were identified, with CMV notably prevalent across samples. Evaluation of viral abundance using viral reads and TPM values revealed CMV dominance in reads and PeMoV prominence in normalized values in select samples. Complete genomes of BCMV, PeMoV, BrYV, and CMV segments were assembled, enabling phylogenetic analysis that uncovered genetic relationships among viral isolates. RT-PCR confirmed BCMV, CMV, and PeMoV presence. Genetic diversity within BCMV was evident through single-nucleotide polymorphism (SNP) analysis, displaying diverse patterns and correlations with viral reads. This study discusses the implications for peanut cultivation, stressing the importance of ongoing research to manage viral diseases. It forms a foundational resource for future investigations into peanut virology, guiding strategies for disease management in peanut crops.

## 1. Introduction

Peanuts (*Arachis hypogaea* L.), known as groundnut, are a global crop, providing both oil and food. Their seeds are rich in monounsaturated fatty acids (50%), promoting vascular health and lowering cholesterol levels [1]. They also offer high-quality protein and various nutrients, supporting fatigue recovery, anti-aging, memory enhancement, immune system strengthening, and blood sugar regulation. Consuming peanuts and nuts is linked to reduced heart disease and cancer risk [2]. They are enjoyed in various forms like roasted, boiled, oil, butter, candy, and chocolate.

In 2016, peanut cultivation in South Korea spanned 5632 hectares, resulting in an estimated yield of 15,500 tons, as reported by the Korean Statistical Information Service. Over the past two decades, peanut production in South Korea has consistently risen, establishing peanuts as a significant legume crop, second only to soybeans. This underscores the need for further research related to peanuts [3]. Peanut cultivation is widespread in South Korea, with major production areas including Yeoju, Yecheon, Gochang, Buan, Jeongeup, Jeju, Seosan, and Taean.

Peanut cultivation faces significant challenges, notably diseases like leaf spot caused by *Cercospora arachidicola* and *Cercosporidium personatum*, as well as groundnut rust caused by *Puccinia arachidis* Speg [4,5]. These diseases are highly destructive in peanut-growing regions, capable of reducing pod yield by up to 50–60%.

In Korea, a total of 13 fungal diseases and five viral diseases affecting peanut plants have been reported according to the List of Plant Diseases in Korea (LPDK). These diverse pathogens pose a continuous threat to peanut cultivation, leading to substantial yield losses due to the necessity for repeated planting. Among the known RNA viruses impacting peanut plants are bean common mosaic virus (BCMV), peanut mottle virus (PeMoV), cucumber mosaic virus (CMV), peanut stunt virus (PSV), and tomato spotted wilt virus (TSWV) [3,6,7,8,9]. Of these viruses, BCMV, belonging to the *Potyvirus* genus, is a prominent virus affecting peanut crops in Korea. Peanut plants infected by BCMV exhibit symptoms like yellowing, mosaic patterns, and stripes, resulting in smaller seed sizes and decreased seed yield [6]. BCMV is known to be transmitted by aphids in a non-persistent manner and through seeds [10,11].

With the rapid advancement of high-throughput sequencing (HTS) technology and bioinformatics tools, it has become feasible to identify both known and novel viruses in various agricultural plants [12,13]. Specifically, HTS-based virome studies offer a wealth of valuable information, including virus detection, viral genome sequences, and viral abundances within a given plant. For instance, in Korea, HTS, followed by bioinformatic analyses, has been employed to unveil the viromes of several significant agricultural plants, including pepper, soybean, sweet potatoes, peach, and barley [14,15,16,17,18].

Despite peanuts being a crucial crop globally, including in Korea, to the best of our knowledge, there has not been an HTS-based virome study conducted for peanuts. In this study, we collected leaves from 15 diverse peanut cultivars and conducted a comprehensive virome analysis.

## 2. Results

### 2.1. Collection of Leaf Samples and Observation of Viral Disease Symptoms in 15 Peanut Cultivars

**Figure 1:**
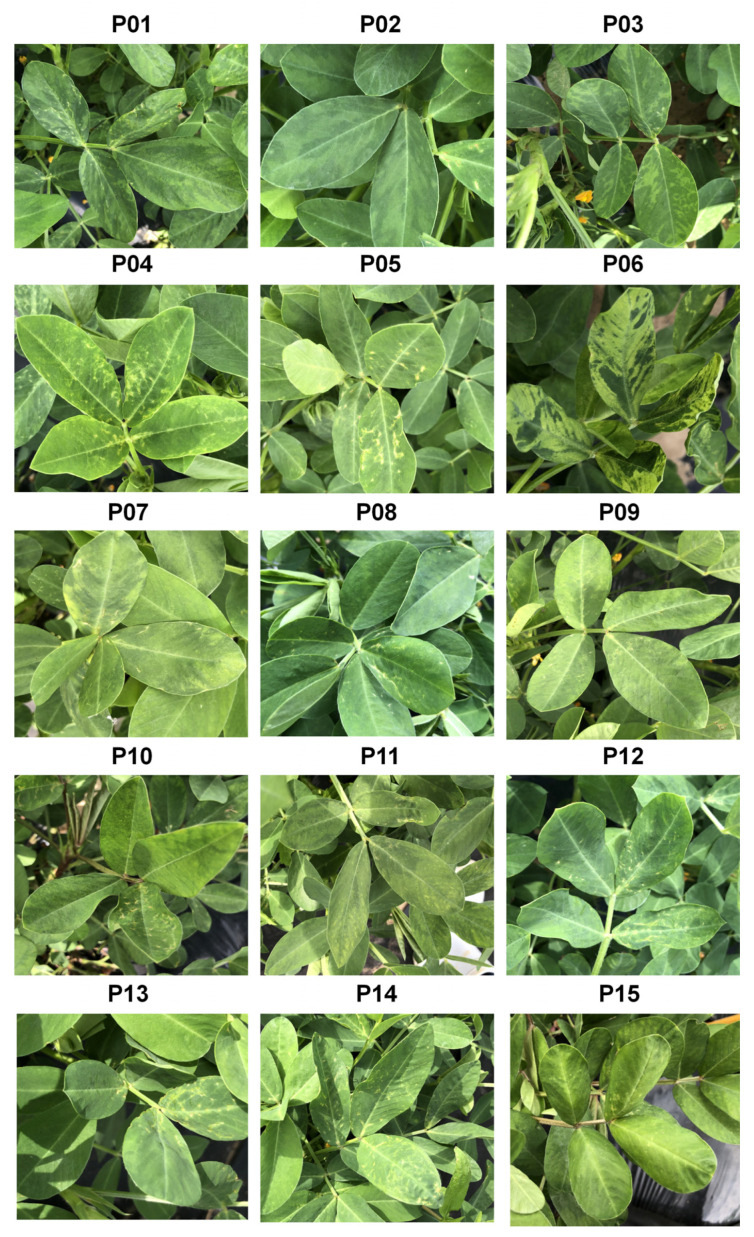
Representative viral disease symptoms in 15 peanut cultivars. Mild to severe foliar symptoms, including mosaic patterns, mottling, chlorosis, stunting, and leaf yellowing, are shown for each cultivar, alongside asymptomatic plants such as P04, P05, and P15.

Fifteen peanut cultivars were grown at the National Institute of Crop Science in Miryang, South Korea (Table 1). Among these cultivars, twelve displayed the typical seed coat colors, while P10 and P15 featured black seed coats, and P12 had a red seed coat (Table 1). It is noteworthy that seed coat color is reported only as basic cultivar metadata and that no statistical analysis was performed to link seed coat color to virus infection or virome composition. These samples showed various symptoms, including mosaic patterns, stunting, and yellowing, as depicted in Figure 1. Such symptoms are known from previous studies to adversely affect plant growth and yield in peanut and other legumes, but in this study we recorded only visible foliar symptoms and did not quantitatively measure growth or yield. Notably, P06 (Sangan) exhibited the most severe disease symptoms, which included mosaic patterns, mottling, chlorosis, and leaf yellowing, whereas the remaining peanut cultivars displayed milder viral disease symptoms or were entirely asymptomatic, such as P04, P05, and P15. Interestingly, cultivar P15 appeared asymptomatic at the time of sampling, even though subsequent RNA-seq analysis revealed a high abundance of CMV reads in this plant.

### 2.2. Identification of Viral Contigs from 15 Different Peanut Cultivars

In our investigation of viruses within 15 selected peanut cultivars, we collected five leaf samples from a single plant representing each individual cultivar. Following total RNA extraction, we prepared ribo-depleted libraries for these 15 cultivars. Subsequent RNA sequencing generated raw data, yielding between 32 million reads (P07) and 47 million reads (P01). We have deposited the raw data in the SRA database at NCBI, and each dataset is associated with specific accession numbers (Table 2).

**Figure 2:**
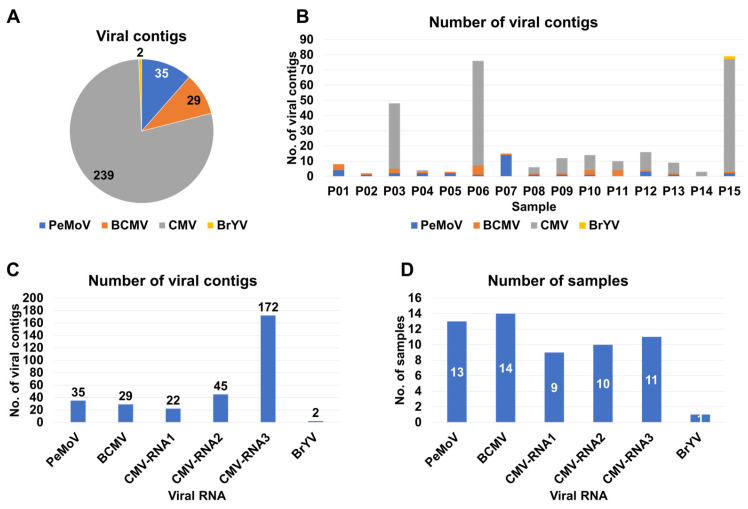
Distribution of the number of identified viral contigs in each sample. (**A**) This pie chart provides an overview of the distribution of the number of viral contigs assigned to each identified virus species. (**B**) The bar chart presents the total number of viral contigs in each individual sample. (**C**) The bar chart visualizes the number of viral contigs corresponding to individual viral genome RNA segments. (**D**) The bar graph illustrates the occurrence of individual viral segments across the samples in which they were identified.

For the identification of viruses, we initiated de novo transcriptome assembly utilizing the Trinity program, which resulted in 15 distinct peanut transcriptomes. Each of these transcriptomes was then subjected to a BLASTX search against the viral protein database to identify viral contigs. These identified viral contigs underwent further scrutiny through BLASTX searches against non-redundant protein databases to eliminate non-viral sequences. Our analysis yielded a total of 305 viral contigs, attributed to four different viruses: CMV (239 contigs), PeMoV (35 contigs), BCMV (29 contigs), and BrYV (2 contigs) (Table S1 and Figure 2A). The number of viral contigs obtained from each sample ranged from two contigs (P02) to 79 contigs (P15). Notably, CMV-associated viral contigs were particularly abundant in three samples, specifically P03, P06, and P15 (Figure 2B).

Subsequently, we explored the distribution of viral contigs according to viral RNA segments. In the case of CMV, which is composed of three RNA segments, RNA3 accounted for the highest number of contigs (172 contigs), followed by RNA2 (45 contigs) and RNA1 (22 contigs) (Figure 2C). We also assessed the occurrence of individual viral segments across the sampled cultivars (Figure 2D). BCMV was identified in 14 of the 15 samples, followed by PeMoV, which was found in 13 samples. Regarding CMV, RNA3 was identified in 11 samples, with RNA2 and RNA1 being present in 10 and nine samples, respectively. BrYV was identified in only one sample (Figure 2D).

### 2.3. Viral Abundance Assessment Using Viral Reads and FPKM Values

**Figure 3:**
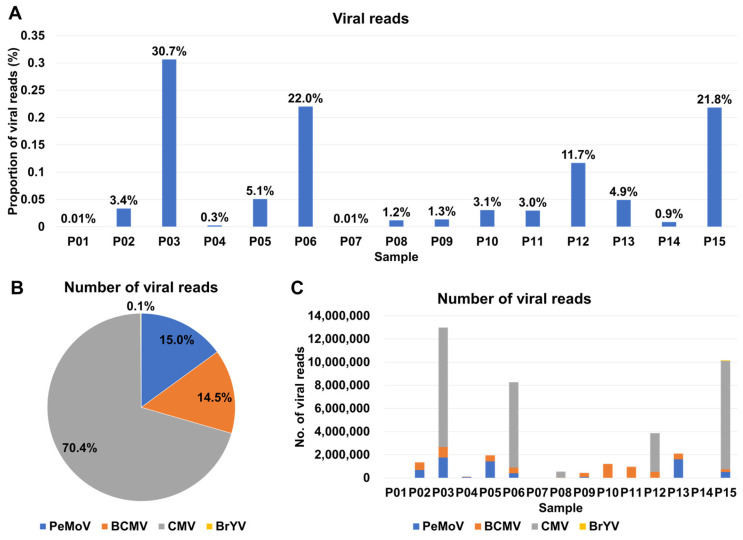
Analysis of viral identification in samples based on viral reads. (**A**) Visualization of the relative proportion of viral reads (highlighted in blue) within the total reads for each sample. (**B**) The ratio of identified viruses concerning the viral reads present in the samples. (**C**) The quantity of viral reads in each individual sample.

Based on the analysis of viral reads, we estimated the proportion of viral reads in each sample, with values ranging from as low as 0.01% (P01 and P07) to as high as 30.7% (P03) (Table S1 and Figure 3A). Notably, in four samples, namely P03, P06, P12, and P15, the proportion of viral reads exceeded 10%. Upon assessing viral abundance using viral reads, we observed that CMV was once again the most prevalent virus, constituting 70.4% of the viral content, followed by PeMoV (15%), BCMV (14.5%), and BrYV (0.1%) (Figure 3B). Furthermore, the number of viral reads varied across samples, with P01, P07, and P14 exhibiting a lower number of viral reads, while P03, P06, P12, and P15, where CMV was notably abundant, displayed a higher number of viral reads (Figure 3C).

**Figure 4:**
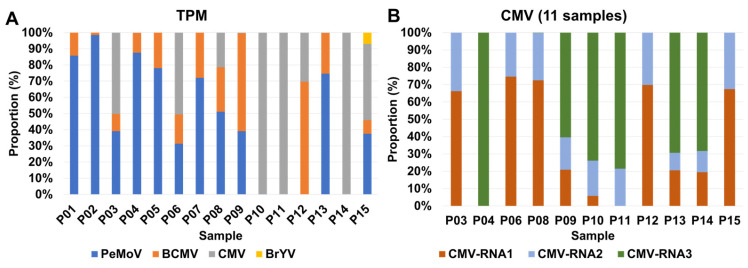
Distribution of identified viruses based on TPM values. (**A**) Relative abundance of identified viruses in each sample using TPM values. (**B**) Relative abundance of three CMV RNA segments in samples with CMV.

To gauge the abundance of identified viruses in each sample, we initially recognized that the number of viral contigs alone might not provide a comprehensive representation of viral abundance. To address this, we turned to viral reads as a more informative metric. Subsequently, we turned our attention to assessing the proportion of viral abundance using TPM (Transcripts Per Million) values, which offer a normalization according to virus genome size (Table S1 and Figure 4). Among the seven samples, namely P01, P02, P04, P05, P07, P08, and P13, PeMoV emerged as the most prevalent virus (Figure 4A). In contrast, CMV took precedence as the most abundant virus in six samples, specifically P03, P06, P10, P11, P14, and P15. It is particularly noteworthy to examine the distribution of individual RNA segments for CMV (Figure 4B). CMV RNA1 stood out as the most abundant segment in five samples, including P03, P06, P08, P12, and P15, while CMV RNA3 claimed this distinction in six samples, encompassing P04, P09, P10, P11, P13, and P14. CMV RNA2 exhibited very low or undetectable levels across samples. This is likely due to segment-specific differences in the accumulation of CMV genomic RNAs in planta and technical bias in the RNA-seq library preparation, which can underestimate segments present at low abundance.

### 2.4. Viral Genome Assembly and Phylogenetic Analysis of Identified Viruses Using Assembled Viral Genomes

Through de novo assembly, we obtained numerous viral contigs that cover the open reading frames (ORFs) of complete viral genomes. Consequently, we successfully acquired complete viral genomes, including 15 BCMV genomes, nine PeMoV genomes, and one BrYV genome (Table S2). For CMV, which consists of three segments, we obtained complete RNA genome sequences for six RNA1, eight RNA2, and six RNA3.

**Figure 5:**
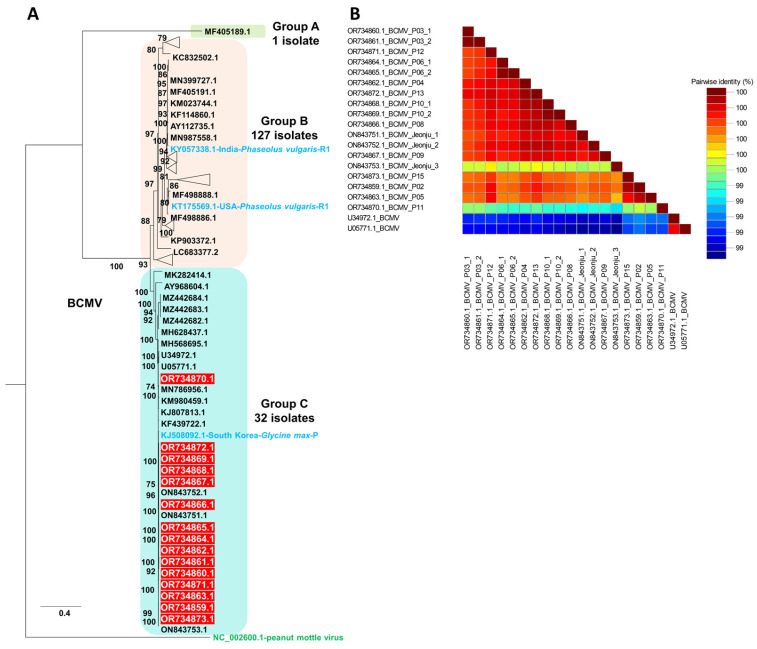
(**A**) Maximum likelihood phylogenetic tree inferred from the complete genome sequences of 160 BCMV isolates, with peanut mottle virus (NC_002600) used as an outgroup, including the 15 Korean BCMV isolates highlighted in red. The tree was reconstructed in IQ-TREE using the GTR+F+R8 nucleotide substitution model selected under the Bayesian Information Criterion and node support was assessed with 1000 ultrafast bootstrap replicates. A triangle was used to collapse and simplify each well-supported cluster of closely related sequences, which are presented at full tip resolution in Supplementary Figure S1. Red boxes indicate BCMV isolates from this study. Light blue shows reference BCMV isolates in non-recombinant (S, P, C) and recombinant (R1, R2, R3) phylogroups, while green denotes peanut mottle virus. (**B**) Pairwise nucleotide identity matrix of complete BCMV genomes generated with the Sequence Demarcation Tool (SDT), showing color-coded pairwise identities among the 15 Korean isolates and closely related reference isolates.

The complete genome-based maximum likelihood tree constructed for 160 BCMV isolates resolved three well-supported clusters corresponding to Groups A, B, and C, with peanut mottle virus (NC_002600) used as an outgroup (Table S3, Figure 5 and Figure S1). Group A contained a single isolate, Group B comprised 127 isolates collected mainly from Phaseolus spp. and Vigna spp., and Group C comprised 32 isolates that included all 15 Korean soybean-derived isolates highlighted in red, which formed a strongly supported monophyletic subcluster together with previously reported soybean-infecting BCMV isolates from South Korea, indicating a close evolutionary relationship and a stable soybean-adapted lineage. In agreement with recent whole-genome comparisons showing that global BCMV isolates segregate into non-recombinant phylogroups S, P and C and recombinant phylogroups R1, R2 and R3 [19], the topology of the complete genome phylogeny indicates that the Korean isolates belong to the established non-recombinant phylogroup C, whereas other reference isolates in Groups A and B include representatives of the recombinant phylogroups R1–R3. Consistently, recombination analyses of the full-length genomes using multiple detection algorithms (RDP, GENECONV, MaxChi and BootScan) did not identify statistically supported recombinant breakpoints within the Korean sequences, whereas several reference isolates in Groups A and B exhibited clear mosaic patterns characteristic of R1–R3, confirming that the Korean BCMV isolates represent a genetically cohesive, non-recombinant lineage within phylogroup C and that their placement is consistent with the current whole-genome phylogroup scheme for BCMV.

The pairwise nucleotide identity matrix generated using SDT showed that the Korean isolates from this study exhibit very high sequence identity to one another and to the Korean reference isolates Jeonju-1, Jeonju-2, and Jeonju-3, whereas they share comparatively lower identity with foreign isolates such as U34972.1-BCMV and U05771.1-BCMV, reflecting the greater genetic divergence of those lineages (Figure 5B).

**Figure 6:**
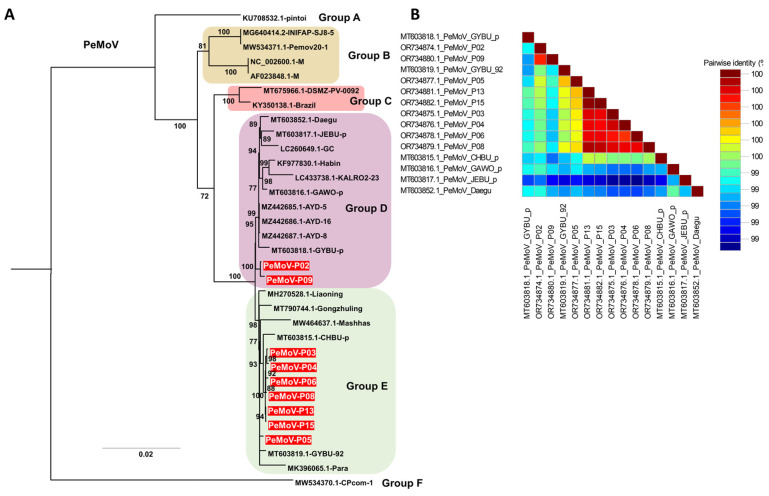
Pairwise nucleotide identity and phylogenetic relationships of PeMoV isolates based on complete genome sequences. (**A**) Maximum likelihood phylogenetic tree inferred from the complete genome sequences of 33 PeMoV isolates, including the nine Korean isolates obtained in this study (red labels). Six major clusters (Groups A–F) were identified, with branch support estimated using 1000 bootstrap replicates under the TIM2+F+G4 substitution model selected by the Bayesian Information Criterion. (**B**) Pairwise nucleotide identity matrix of PeMoV complete genomes generated using SDT, showing sequence similarity among nine Korean field isolates and closely related reference isolates from Korea and other countries.

Phylogenetic analysis of the 33 PeMoV isolates, comprising 24 previously reported genomes and nine genomes obtained in this study, resolved six distinct clusters (Groups A–F) (Table S4, Figure 6A). Within Group F, the CPcom-1 isolate from *Phaseolus vulgaris* in Zambia formed a long, isolated branch, indicating marked genetic divergence from the remaining PeMoV isolates, while the pintoi isolate from *Arachis pintoi* in Colombia in Group B occupied a basal position that suggests it may represent an ancestral lineage relative to the other 31 isolates. Groups D and E were the largest clusters, containing 12 and 13 isolates, respectively, and both included multiple soybean-derived PeMoV isolates from Korea; among the isolates generated in this study, two were assigned to Group D and seven to Group E, where they formed well-supported subclades with Korean reference isolates. The pairwise nucleotide identity matrix indicated that the nine PeMoV isolates from this study shared high nucleotide identity with each other and with previously reported Korean PeMoV genomes, whereas their identities with foreign isolates, such as those from Zambia and Colombia, were comparatively lower, reflecting greater genetic divergence among geographically distant lineages (Figure 6B).

**Figure 7:**
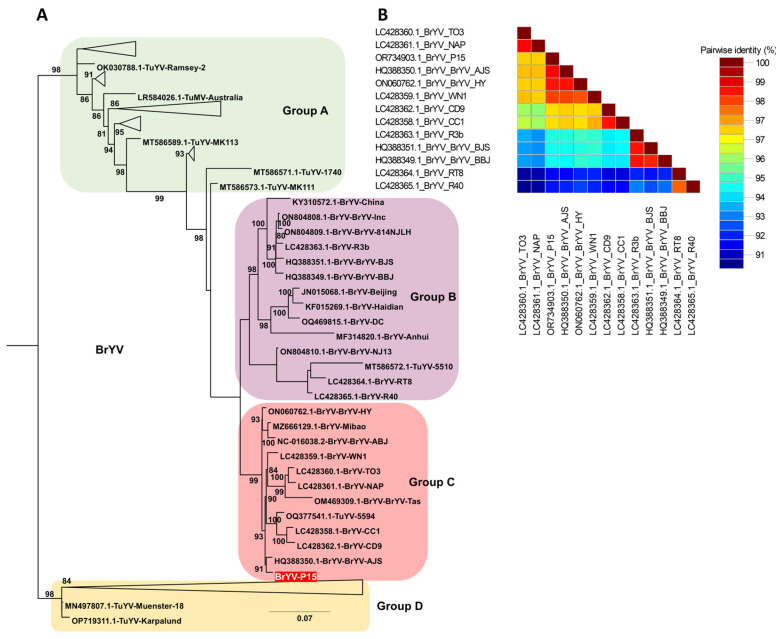
Phylogenetic relationships and pairwise nucleotide identity of BrYV and TuYV isolates based on complete genome sequences. (**A**) Maximum likelihood phylogenetic tree inferred from 100 complete genomes of brassica yellows virus (BrYV) and turnip yellows virus (TuYV), including one BrYV isolate (BrYV-P15, red label) obtained in this study. Four major groups (A–D) were resolved, and branch support was calculated using 1000 bootstrap replicates under the TIM2+F+I+G4 substitution model selected by the Bayesian Information Criterion. A triangle was used to collapse and simplify clustered groups, which are shown in full detail in Supplementary Figure S2. (**B**) Pairwise nucleotide identity matrix of representative BrYV and TuYV genomes generated using SDT, illustrating the sequence similarity between the BrYV-P15 isolate and closely related BrYV and TuYV reference isolates.

Based on BLASTN analysis, many BrYV isolates showed high sequence similarity to TuYV, prompting construction of a combined dataset comprising all available BrYV and TuYV genomes together with the BrYV-P15 genome generated in this study. Comprehensive phylogenetic analysis of these 100 genomes resolved four distinct groups (A–D) (Table S5, Figure 7A and Figure S2). Group A consisted mainly of TuYV, containing 30 isolates in total, of which 28 were TuYV and two were BrYV (Cheongsong and 21706265). Group B included 14 isolates (13 BrYV and one TuYV, isolate 5510), whereas Group C comprised 12 isolates (11 BrYV and one TuYV, isolate 5594), with BrYV-P15 from this study clustering within Group C. Group D was the largest cluster, containing 44 isolates, including 41 TuYV genomes and three BrYV isolates (1-1, BrYV-HQ, and BrYV-NtabQJ), reflecting extensive intermixing of BrYV and TuYV lineages in this group. The pairwise nucleotide identity matrix confirmed the close relationship between BrYV-P15 and BrYV/TuYV isolates belonging to Group C, which shared higher identity values with BrYV-P15 than with more distantly related isolates from Groups A and D, consistent with their clustering pattern in the phylogenetic tree (Figure 7B).

**Figure 8:**
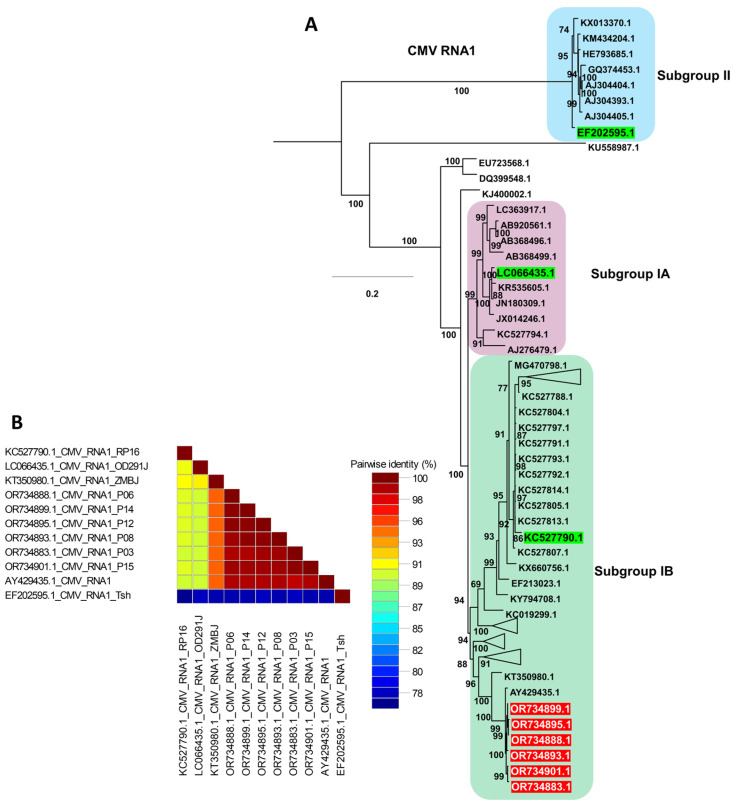
Phylogenetic analysis and pairwise nucleotide identity of CMV RNA1 isolates. (**A**) Maximum likelihood phylogenetic tree of CMV RNA1 constructed using available complete genome sequences, including the isolates obtained in this study (highlighted in red). The tree was inferred with 1000 bootstrap replicates under the GTR+F+I+G4 substitution model selected by the Bayesian Information Criterion. Green-labeled reference isolates represent previously characterized members of subgroups IA, IB, and II described in earlier studies; to avoid redundancy, only these representative isolates are shown, and the full phylogenetic trees are not presented. (**B**) Pairwise nucleotide identity matrix of selected CMV RNA1 genomes generated using SDT, illustrating the high sequence similarity among the RNA1 isolates from this study and their closest subgroup IB reference isolates.

For the CMV phylogenetic analysis, only isolates with complete genome sequences for all three RNA segments were included, together with the six CMV isolates obtained in this study (Table S6). Phylogenetic analysis of CMV RNA1 resolved several distinct groups (Figure 8A), with subgroup II forming a basal lineage that appears ancestral to the remaining CMV isolates, consistent with previous reports [20]. All six CMV RNA1 sequences from this study (red labels) clustered within subgroup IB, forming a tightly supported clade that grouped closely with Korean reference isolates as well as the ZMBJ isolate from maize in China and the CS isolate from *Arachis hypogaea* L. in China, indicating a close genetic relationship among these subgroup IB members (Table S6 and Figure 8A). Representative green-labeled isolates from subgroups IA, IB, and II were included as anchors based on earlier classification studies, and only these were displayed to simplify the tree; the full RNA1 phylogeny is therefore not shown. Consistent with the tree topology, the pairwise nucleotide identity matrix (Figure 8B) showed high nucleotide identity among the RNA1 sequences from this study and their subgroup IB reference isolates, whereas noticeably lower identity values were observed in comparisons with representative subgroup IA and II genomes, supporting greater genetic divergence between these subgroups.

**Figure 9:**
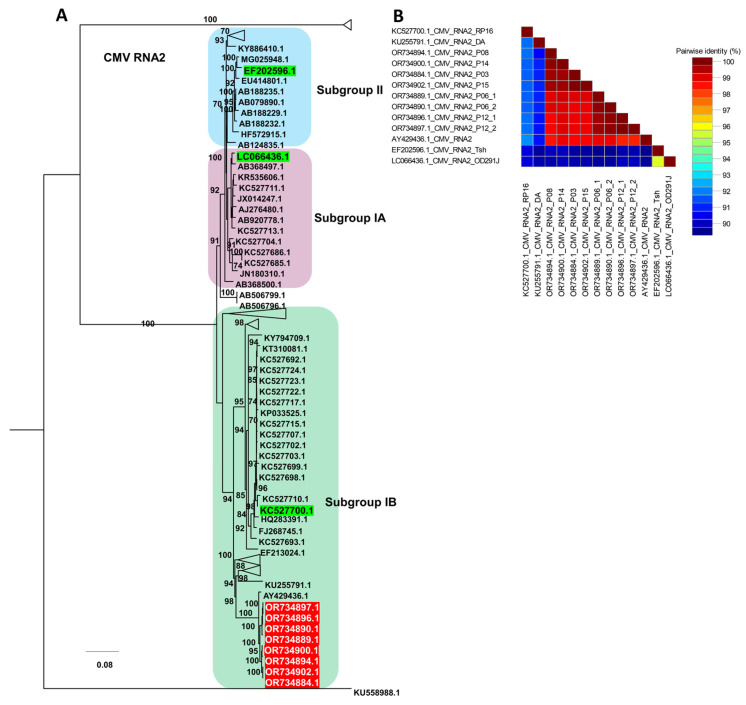
Phylogenetic analysis and pairwise nucleotide identity of CMV RNA2 isolates. (**A**) Maximum likelihood phylogenetic tree of CMV RNA2 constructed using available complete genome sequences, including the six isolates obtained in this study (highlighted in red). The tree was inferred with 1000 bootstrap replicates under the TIM2+F+I+G4 substitution model selected by the Bayesian Information Criterion; subgroup IA, IB, and II reference isolates previously defined for CMV are shown in green, and only representative sequences are displayed to simplify the topology. (**B**) Pairwise nucleotide identity matrix of selected CMV RNA2 genomes generated using SDT, illustrating sequence similarity among the RNA2 isolates from this study and their closest subgroup IB reference isolates.

For CMV RNA2, the phylogenetic tree resolved three main subgroupings corresponding to subgroups IA, IB, and II, with representative reference isolates for each subgroup highlighted in green (Table S7 and Figure 9A). All RNA2 sequences from this study (red labels) clustered within subgroup IB, forming a tightly supported clade together with several previously reported subgroup IB reference isolates, indicating close genetic relatedness within this lineage. The pairwise nucleotide identity matrix showed uniformly high identity values among the six RNA2 isolates from this study and their closest subgroup IB references, whereas identity values were lower when these isolates were compared with representative subgroup IA and II genomes, reflecting greater divergence between subgroups (Figure 9B).

**Figure 10:**
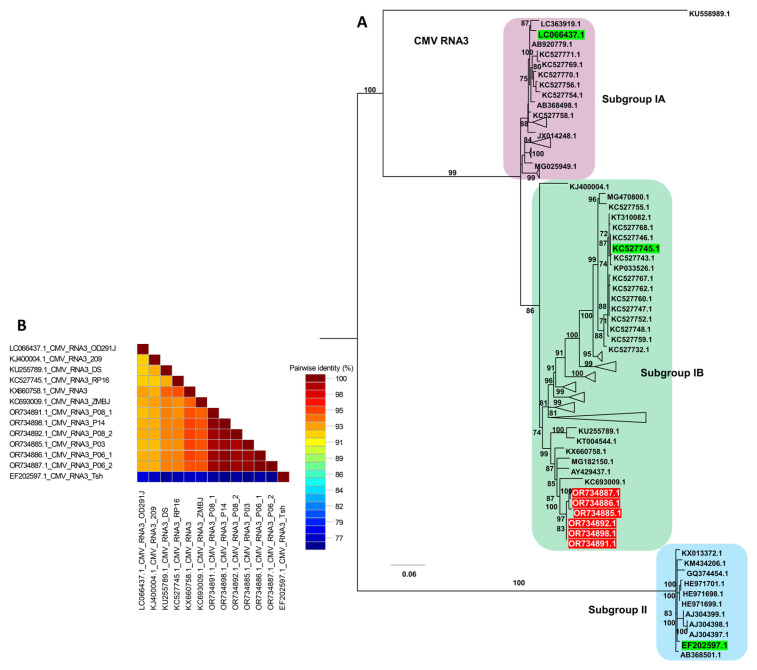
Phylogenetic analysis and pairwise nucleotide identity of CMV RNA3 isolates. (**A**) Maximum likelihood phylogenetic tree of CMV RNA3 constructed using available complete genome sequences, including the six isolates obtained in this study (highlighted in red). The tree was inferred with 1000 bootstrap replicates under the TIM3e+I+G4 substitution model selected by the Bayesian Information Criterion; representative reference isolates for subgroups IA, IB, and II are shown in green, and only these are displayed to simplify the topology. (**B**) Pairwise nucleotide identity matrix of selected CMV RNA3 genomes generated using SDT, illustrating sequence similarity among the RNA3 isolates from this study and their closest subgroup IB reference isolates.

Phylogenetic analysis of CMV RNA3 resolved seven distinct groups (Table S8 and Figure 10A). Similar to the pattern observed for RNA1, isolates within Group A occupied a basal position and appeared ancestral relative to the other RNA3 lineages. Group F contained all six RNA3 sequences from this study, which clustered tightly with several previously reported isolates, including Anhui, ZMBJ, K-2016, CS, DS, WN1, and 209, indicating a close genetic relationship among these Group F members. Consistent with the tree topology, the pairwise nucleotide identity matrix showed high nucleotide identity among the six RNA3 isolates from this study and their Group F reference isolates, whereas lower identity values were observed when these isolates were compared with more distantly related genomes from other groups (Figure 10B).

### 2.5. Confirmation of RNA Sequencing Results by RT-PCR

**Figure 11:**
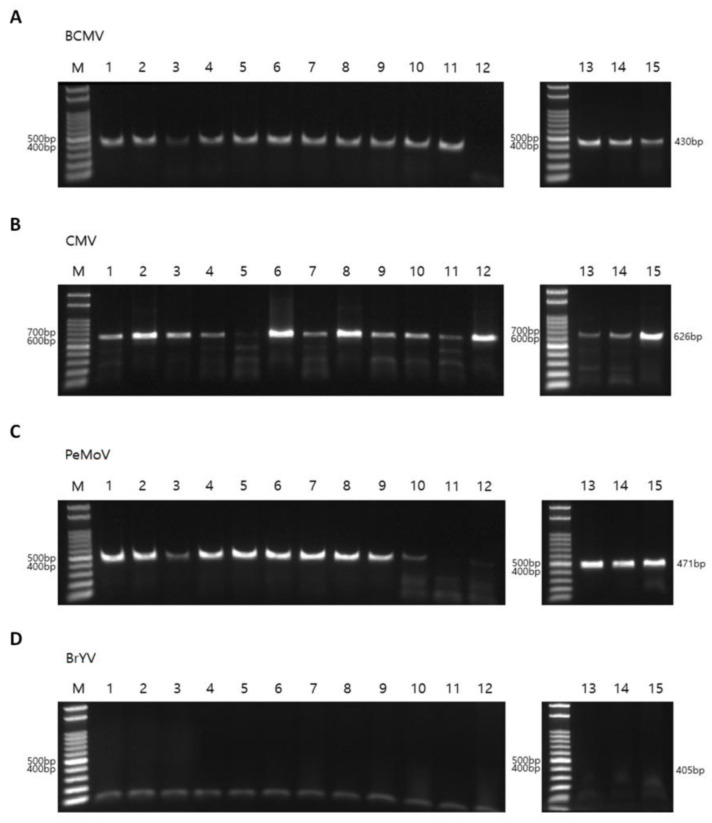
Detection of viruses infecting peanut plants by RT-PCR. RT-PCR results using virus specific primers for BCMV (**A**), CMV (**B**), PeMoV (**C**), and BrYV (**D**). PCR products were electrophoresed on 1.5% agarose gel. The same total RNAs used for RNA sequencing were used for RT-PCR. M indicates DNA markers. The expected amplicon size for each virus were indicated on the right.

To validate the RNA sequencing results, RT-PCR was performed using virus-specific primers, including four newly designed primer pairs (Table 3). The RT-PCR primers for BCMV, CMV, PeMoV, and BrYV were designed based on consensus regions of the viral genomes assembled from RNA-seq data in this study, supplemented by alignment with representative GenBank reference sequences to ensure broad specificity and minimize possible primer–template mismatches. Using BCMV-specific primers and the same total RNAs used for RNA sequencing, BCMV was detected by RT-PCR in 14 of the 15 peanut samples (all except P12; Figure 11A and Figure S3). In contrast, RNA sequencing additionally revealed BCMV reads and enabled assembly of a complete BCMV genome from P12. Using CMV-specific primer pairs, CMV was detected in all peanut samples except P05 (Figure 11B). PeMoV infection was detected in 13 peanut samples by RT-PCR, except P11 and P12 (Figure 11C). In addition, RT-PCR using primer pairs for BrYV—which was not identified by RNA sequencing—showed that none of the peanut samples was infected by BrYV (Figure 11D). Finally, RT-PCR assays for TSWV indicated that none of the samples was infected by TSWV (Figure S3).

### 2.6. Analysis of Single-Nucleotide Polymorphisms in BCMV in Across Samples

**Figure 12:**
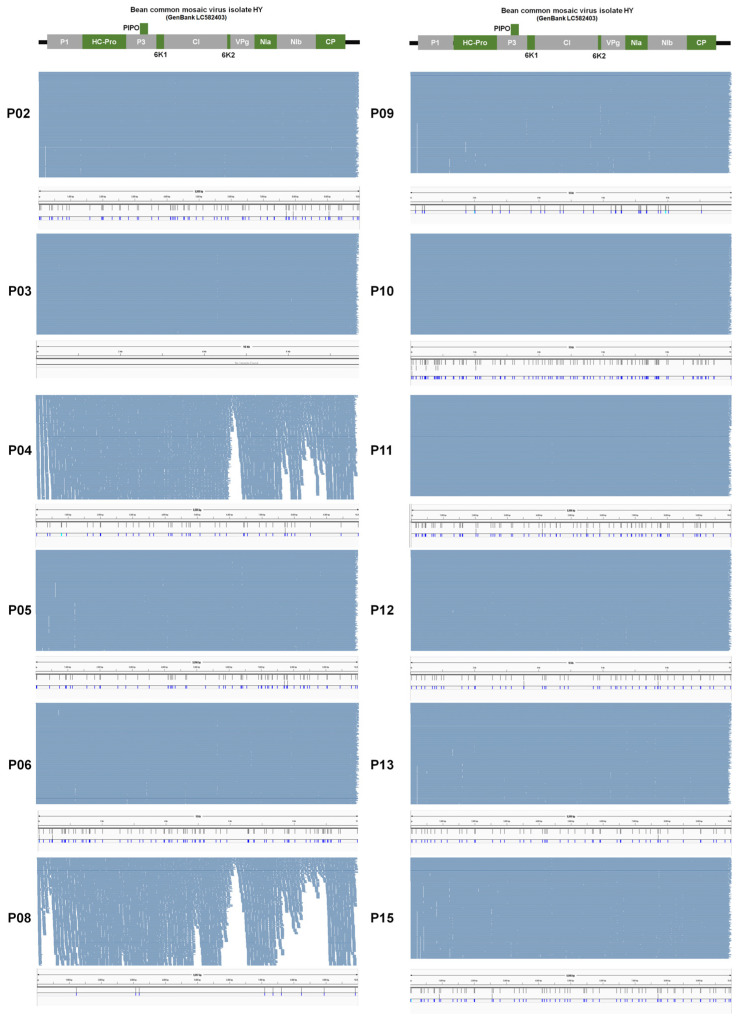
Visualization of the mapping results of raw data onto the BCMV genome and the identification of single-nucleotide polymorphisms (SNPs). To uncover BCMV SNPs within each sample, we employed the assembled BCMV genome from each sample as a reference. Raw data was then aligned with the BCMV reference genome, and subsequent SNP analysis was conducted. Each sample is represented by two distinct images. The upper image depicts the alignment of raw data onto the BCMV genome, while the bottom image highlights the positions of identified SNPs on the BCMV genome.

**Figure 13:**
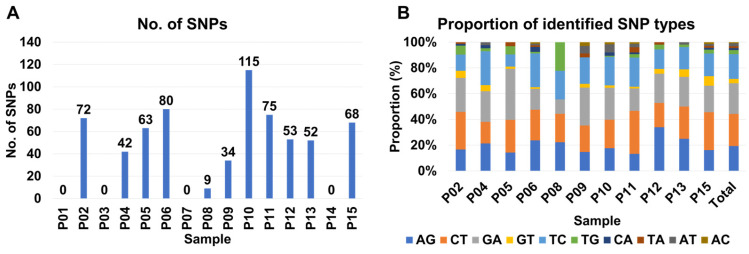
Analysis of SNP of BCMV in each sample. (**A**) Number of identified SNPs and (**B**) proportion of identified SNP types in each sample. AG indicates the conversion of adenine (A) to guanine (G), while CT indicates the conversion of cytosine (C) to thymine (T) in the single-nucleotide polymorphisms (SNPs).

From some samples such as P03, P06, and P10, at least two different BCMV isolates were present suggesting quasispecies of BCMV in the infected sample. We examined single-nucleotide polymorphisms (SNPs) of BCMV in each sample to reveal genetic diversity and variation of BCMV genomes within the infected plant sample. For that, raw data were mapped on the BCMV genome which was obtained from the same sample and SNPs were analyzed. As shown in Figure 12, numerous BCMV-associated reads were mapped on the BCMV genome. We did not get any SNPs for the P01, P03, and P07 samples (Figure 13A). Notably, P02, P05, P06, and P15 exhibit relatively high SNP counts (72, 63, 80, and 68, respectively), while P10 and P11 show elevated counts (115 and 75) (Figure 13A). Moderately counted SNPs (ranging from 34 to 53) are observed in P04, P09, P12, and P13.

Additionally, the SNP analysis reveals diverse patterns, with A to G, C to T, and G to A showing moderate to high occurrences, while G to T, T to G, and C to A exhibit lower frequencies (Figure 13B). Total counts for these SNP types range from 10 to 165 across samples, providing insights into the genetic diversity in the dataset. Furthermore, a correlation matrix among variables indicates a moderate positive correlation (0.665) between SNP counts and viral reads, a weaker positive correlation (0.280) between SNPs and viral contigs, and a moderate positive correlation (0.395) between viral reads and contigs.

## 3. Discussion

Our study delves into the peanut virome in Korea, a critical exploration considering the mounting risks posed by new viral diseases in field crops due to global warming and climate change [21]. While extensive research has targeted viral diseases affecting diverse field crops, there has been a noticeable gap in peanut virome studies. Our investigation aims to fill this void by analyzing the viromes of 15 distinct peanut cultivars. The findings offer valuable insights into the current viral landscape of peanuts in the surveyed region and provide a basis for future monitoring of peanut viruses in agricultural production systems.

Our study utilized HTS and RT-PCR to identify viruses in 15 distinct peanut cultivars, revealing the presence of three major viruses—BCMV, CMV, and PeMoV. In addition, BrYV-like sequences were detected by HTS and a BrYV-like genome was assembled from peanut, providing preliminary sequence-based evidence of a BrYV-related virus associated with peanut in Korea, but this finding remains unvalidated by RT-PCR and should be interpreted with caution. Similarly, previous studies investigating viromes in *Arachis* species have identified at least three to four viruses. For instance, in Turkey, 63 peanut samples underwent RT-PCR analysis, revealing the presence of PeMoV, peanut stunt virus (PSV), and TuYV [22]. Furthermore, a virome study on forage peanut (*Arachis pintoi*) conducted via HTS identified PeMoV, TuYV, and two allexiviruses in Colombia [23].

One aim of this study was to describe differences in virus accumulation among individual peanut plants representing 15 cultivars under field conditions, rather than to determine definitive cultivar resistance or susceptibility. To this end, viral abundance in each sample was quantified using two metrics: the number of viral reads and TPM values. The viral read count for each sampled plant reflected relative viral accumulation; for example, P03 (30.7%), P06 (22%), and P15 (21.8%) exhibited high proportions of viral reads for all three viruses, indicating a relatively high viral load compared with plant viromes reported in other crops such as soybean [15,24]. Notably, these plants showed coinfection by all three viruses, with CMV being the dominant virus. In contrast, the Gowon plant (P14) exhibited only CMV infection with low abundance, suggesting lower accumulation of BCMV and PeMoV in that sampled plant, whereas the Heukchan plant—a medium-sized grain cultivar with a dark-purple seed coat developed in 2019 at the Department of Southern Area Crop Science, National Institute of Crop Science in Korea—showed high levels of all three viruses [25]. These field-based patterns of viral load in individual plants should therefore be regarded as preliminary indicators that can help select candidate cultivars for future experiments using multiple biological replicates, randomized field designs and/or controlled inoculations, and appropriate statistical analyses to rigorously evaluate viral disease resistance in peanut.

Purely based on the frequency of infected viruses, BCMV could be perceived as a prevalent virus affecting peanut plants in Korea. However, when considering the TPM value, a normalized measure of viral composition, PeMoV emerged as dominant in at least seven samples, while CMV took precedence in six samples. These results indicate that different viruses can reach high titers in mixed infections, but they do not allow us to attribute specific symptom types or severities to individual viruses in coinfected plants. BCMV, PeMoV, and CMV are important legume-infecting viruses that can co-occur in the same plants or fields [26], but the precise contribution of each virus or virus combination to symptom expression will need to be clarified in future studies using artificial inoculation experiments with single and mixed infections under controlled conditions.

Likewise, we investigated the proportion of three CMV RNA segments in each sample, revealing RNA1 dominance in five samples and RNA3 dominance in six samples. This finding indicates a notably higher viral abundance for RNA1 or RNA3 compared to RNA2, potentially influenced by the specific peanut cultivar. A previous study similarly noted elevated levels of CMV RNA1 or RNA3 in various soybean cultivars [24]. This hints at the likelihood of viral abundance for the three CMV RNA segments being contingent upon the plant species and its environmental conditions.

One advantage of HTS techniques in virome analysis might be the acquisition of complete viral genomes. In comparison to other similar studies, we obtained a significant number of viral genomes (15 BCMV, 9 PeMoV, 1 BrYV, and 20 CMV) from a single study. Due to the benefits of ribosome-depleted libraries, we could obtain viral genomes from diverse species, such as two kinds of potyviruses with poly(A) tails, as well as two viruses without poly(A) tails, such as a polerovirus and a cucumovirus. We believe that the plant materials play an important role in obtaining complete viral genomes through HTS. In our study, most peanut plants showed mild symptoms in the harvested leaves, which could be suitable material for detecting virus infection in peanuts.

A phylogenetic analysis, utilizing 160 BCMV genomes, including 15 genomes from this study, unveiled 10 distinct groups. In comparison to a recent study that conducted phylogenetic analysis using 86 BCMV genomes [27], nearly twice the number of viral genomes were employed for this analysis. All 15 BCMV isolates from our study were classified into group C, indicating genetic similarity among Korean BCMV isolates in peanuts. Furthermore, we observed at least two different BCMV isolates in some peanut plants, suggesting the presence of BCMV quasispecies—a collection of genetically diverse viral populations [28]. It is understood that the quasispecies of plant RNA viruses are regulated by host–virus interactions [29]. To uncover the genetic diversity within BCMV populations, we examined SNPs for BCMV, revealing a moderate positive correlation (0.665) between SNP counts and viral reads (abundance). Additionally, we identified common conversions, such as A to G, C to T, G to A, and T to C, within the BCMV genome. These frequently identified conversions were also recognized in other potyviruses, like SMV in a previous study [15]. It appears that these common conversions might be conserved across potyviruses.

PeMoV induces mottling, mosaic patterns, and yellowing symptoms in infected samples, affecting not only peanuts but also soybeans, French beans, and peas [30]. In our study, we sequenced nine PeMoV genomes, categorized into two groups: Group D (comprising two isolates) and Group E (consisting of seven isolates). This finding indicates distinct genetic variations within PeMoV. Group D notably encompassed several Korean PeMoV isolates from soybeans, suggesting possible transmission between soybean and peanut, contributing to the genetic diversity of PeMoV. CMV exhibits a broad spectrum of plant hosts and stands as the most frequently identified virus in various crop plants across Korea. Our phylogenetic analysis revealed a close relationship among all CMV isolates studied, indicating a high level of sequence similarity among these CMV isolates.

While infections of BCMV, PeMoV, and CMV in peanut plants have been well documented, infection by BrYV in peanut has not been reported so far to our knowledge. In this study, a complete BrYV genome was assembled from peanut, and the BrYV signal in the HTS data was supported by contiguous viral contigs and mapped reads with high sequence similarity to BrYV/TuYV reference genomes, suggesting that BrYV-like sequences were present in the sample. However, BrYV could not be detected by RT-PCR, and several factors may explain this discrepancy. BrYV is a phloem-limited polerovirus, so its titer in bulk leaf tissue may be low and unevenly distributed, while deep sequencing can still recover low-abundance viral RNA. In addition, the RT-PCR primers were designed from available BrYV sequences, and the documented genetic diversity among BrYV/TuYV-like isolates means that primer–template mismatches, particularly near the 3′ termini, may have reduced amplification efficiency and produced false-negative RT-PCR results.

Technical factors may also have contributed: although the same RNA preparations were used for library construction and RT-PCR, RNA degradation during storage or repeated freeze–thaw cycles could disproportionately affect low-titer viruses such as BrYV. Moreover, in mixed BrYV/TuYV populations, close sequence relatedness can occasionally lead to over-assignment of reads to BrYV despite stringent mapping parameters. Taken together, these considerations indicate that, at present, BrYV detection in peanut in this study is supported only by HTS data, and that the failure of RT-PCR likely reflects a combination of low viral titer and sequence divergence from the primer targets. Future work using redesigned primers targeting conserved regions and additional biological replicates, ideally combined with controlled inoculation, will be required to definitively validate BrYV infection in peanut.

The phylogenetic tree including TuYV/BrYV sequences indicated that the peanut BrYV isolate clusters within groups containing TuYV-like isolates, consistent with BrYV being a strain within the TuYV species rather than a distinct species. Reports of TuYV infection in peanuts from Turkey and in forage peanut in Colombia support the conclusion that TuYV/BrYV-like poleroviruses can naturally infect *Arachis* species, including cultivated peanut [22,23]. Poleroviruses like BrYV and TuYV are known to be transmitted by phloem-feeding aphids in a circular manner [31]. Furthermore, potyviruses such as BCMV and PeMoV are transmitted non-persistently and non-circulatively by aphids [32], and they can also spread through seeds [3]. Similarly, CMV is a seed-borne virus transmitted non-persistently by diverse aphids [33]. These well-established transmission modes, reported in previous studies, highlight the general importance of using virus-free seed and implementing aphid management for peanuts and other seed-propagated crops such as soybean; however, our study did not directly evaluate seed or aphid-mediated transmission, and these implications should be considered as background context rather than conclusions drawn from our data [15].

Our study not only advances the understanding of peanut viromes in Korea but also has practical implications for peanut cultivation. The identification of novel viruses like BrYV highlights the dynamic nature of viral populations and the challenges they pose to crop management. Continuous monitoring and research are essential to develop strategies that mitigate the impact of viral diseases on peanut crops. This study sets the stage for future research on the occurrence, genetic diversity, and dynamics of peanut viruses, particularly considering changing environmental conditions.

## 4. Materials and Methods

### 4.1. Investigation of Viral Disease Symptoms and Peanut Leaf Sample Collection

In July 2020, an in-depth examination of viral disease symptoms was carried out on 15 distinct peanut cultivars cultivated at the National Institute of Crop Science in Miryang, South Korea. To scrutinize viruses affecting peanut plants, leaf samples were acquired on 1 July 2020. Additionally, an evaluation of seed coat colors in the peanut cultivars was carried out. Mild viral disease symptoms, such as mosaic patterns, stunting, and yellowing, were observed in the majority of peanut cultivars in their leaf samples. Post-harvest, the peanut leaves were promptly flash-frozen using liquid nitrogen and stored at −80 °C.

### 4.2. Total RNA Extraction from Harvested Peanut Leaf Samples

The frozen peanut leaf samples were finely powdered using a mortar and pestle while maintaining the temperature with liquid nitrogen. Total RNA was then extracted from approximately 100 mg of this ground powder using the RNeasy Plant Mini Kit (Qiagen, Hilden, Germany), following the manufacturer’s guidelines. The extracted total RNAs were preserved at −80 °C for subsequent analysis. The quality of the extracted total RNAs was assessed using the Technologies 2100 Bioanalyzer (Agilent, Santa Clara, CA, USA), and those with an RNA Integrity Number (RIN) equal to or greater than 7 were chosen for library preparation. To prepare the libraries for RNA sequencing, ribosomal RNAs were depleted from the total RNAs using the TruSeq Stranded Total RNA with a Ribo-Zero Plant kit (Illumina, San Diego, CA, USA), following the manufacturer’s instructions. Consequently, 15 libraries, representing the 15 peanut cultivars, were created and labeled as P01–P15.

### 4.3. RNA Sequencing and De Novo Transcriptome Assembly

The 15 libraries underwent paired-end sequencing (150 bp × 2) using the Illumina Nova6000 system. The raw data from these sequencing runs were deposited in the Sequence Read Archive (SRA) database at the National Center for Biotechnology Information (NCBI) and assigned respective accession numbers (SRR26329861 to SRR26329875). The raw data, available as FASTQ files for each library, were utilized for de novo transcriptome assembly using the Trinity assembler (version 2.13.2) with default parameters as outlined previously [34].

### 4.4. Identification of Viral Contigs and Estimation of Viral Abundance

The contigs obtained underwent a BLASTX search against the plant viral database derived from the NCBI (https://www.ncbi.nlm.nih.gov/genome/viruses/ (accessed on 1 June 2023)) with an E-value cutoff of 1 × 10^−10^. To exclude non-viral sequences, the contigs associated with viruses were further subjected to a BLASTX search against the NCBI non-redundant (NR) protein database. Consequently, solely virus-associated contigs were retained from each library. To align the raw sequence reads to the reference viral genomes, we employed the BWA aligner version 0.7.17 with default parameters [35]. The coverage, viral reads, and transcripts per million (TPM) for each virus in individual samples were then calculated using the eXpress 1.5.1 program (https://pachterlab.github.io/eXpress/manual.html) (accessed on 1 June 2023) based on the SAM file.

### 4.5. Assembly of Viral Genome and Virus Genome Annotation

Viral sequences encompassing all open reading frames (ORFs) for each virus species were meticulously selected. These sequences were subjected to the NCBI ORFfinder (http://www.ncbi.nlm.nih.gov/orffinder/) (accessed on 1 June 2023). Each individual ORF in the viral genomes was annotated by referencing known viral genome sequences. The assembled viral genomes were named according to their respective libraries, and in some cases, multiple viral genomes from the same library were identified. All assembled viral genomes were deposited in the NCBI GenBank database, each assigned a unique accession number.

### 4.6. Phylogenetic and Pairwise Sequence Identity Analyses of Viral Genomes

For each virus species, the BLASTN analysis was applied to the assembled viral genomes against the NCBI nucleotide database. Extracted were the known viral genome sequences for each identified virus species, and these were aligned with the viral genome sequences obtained in this study utilizing MAFFT version 7 with the auto option [36]. Following alignment, automated sequence trimming was carried out using TrimAl with the automated1 option [37]. The next step involved importing the trimmed sequences into IQ-TREE version 2.0, employing the maximum likelihood method with 1000 bootstrap replicates [38]. The resulting phylogenetic trees underwent visualization and editing using Figtree version 1.4.4 (http://tree.bio.ed.ac.uk/software/figtree/) (accessed on 1 June 2023). The maximum likelihood trees were inferred and interpreted as unrooted trees, because our primary objective was to examine clustering and genetic relatedness among isolates within each virus species, rather than to reconstruct the directionality of evolution using an explicit outgroup.

Sequence similarity among viral genomes was assessed using the Sequence Demarcation Tool (SDT) v1.3 [39]. For each virus species or genomic segment, nucleotide sequences were imported in FASTA format and all pairwise comparisons were performed under the default pairwise-alignment option, which uses a Needleman–Wunsch global alignment algorithm (as implemented in MUSCLE) to align every unique sequence pair.

### 4.7. Primer Design for Virus Detection in Peanut Plants by RT-PCR

Primer pairs for reverse transcription polymerase chain reaction (RT-PCR) were designed based on the viral genome sequences obtained for four specific viruses: BCMV, CMV, PeMoV, and BrYV (Table 2). Following RT-PCR, the primer pairs that specifically amplified the target virus species were selected. The amplicon sizes ranged from 405 bp to 626 bp. RT-PCR was performed using the AccessQuick™ RT-PCR System Master Mix (2×) (Promega, Madison, WI, USA). The RT-PCR reaction mixtures included 1 µL of extracted total RNA (100 ng), 0.5 µL of the forward primer (10 pmol/µL), 0.5 µL of the reverse primer (10 pmol/µL), 12.5 µL of AccessQuick™ RT-PCR System Master Mix (2×), and 0.5 µL of AMV reverse transcriptase, adjusted to a final volume of 25 µL using RNase-free water. The RT-PCR reaction conditions consisted of cDNA synthesis at 45 °C for 45 min, initial denaturation at 95 °C for 2 min, denaturation at 95 °C for 30 s, annealing at 56 °C for 30 s (for BCMV), annealing at 58 °C for 1 min (for CMV and PeMoV), annealing at 56 °C for 1 min (for BrYV), and extension at 72 °C for 40 s. The denaturation-to-extension steps were repeated 34 times, with a total of 35 cycles, concluding with a final extension step at 72 °C for 5 min.

### 4.8. Single-Nucleotide Polymorphism (SNP) Analyses for BCMV

To pinpoint mutation positions in BCMV, we conducted single-nucleotide polymorphism (SNP) analyses. The identification of exact mutation positions involved utilizing assembled virus genome sequences in each library as reference virus genome sequences. For instance, the assembled viral genomes for BCMV-P02 served as reference genomes for the P02 library. The SNP analysis procedure followed previous methodologies [15]. In summary, we aligned raw sequence reads to the assembled viral genome using the BWA program with default parameters. The resulting sequence alignment map (SAM) files were converted to binary alignment map (BAM) files using the SAMtools program (v.1.17) [40]. Subsequently, the sorted BAM files were transformed into variant call format (VCF) files using the mpileup function of SAMtools. Finally, SNP calling was executed using BCFtools implemented in SAMtools. The Tablet program was employed to visualize the positions of identified SNPs [41].

## 5. Conclusions

In this study, we illuminated a diverse viral landscape affecting 15 peanut cultivars in South Korea. Among the prevalent viruses—CMV, PeMoV, and BCMV—a novel virus, BrYV, was identified, and each showcased varied prevalence and impacts on plant health. This viral abundance showed significant variability across cultivars, with CMV dominating in reads, PeMoV demonstrating prominence in normalized values, and BCMV displaying notable genetic diversity within infected plants. The acquisition of complete viral genomes provided crucial genetic insights, highlighting relationships among isolates and suggesting potential transmission pathways, notably evident in PeMoV and BCMV isolates. RT-PCR validation confirmed BCMV, CMV, and PeMoV presence, corroborating the RNA sequencing findings. Our findings underscore the importance of ongoing research in understanding viral dynamics, genetic diversity, and the need for robust management strategies in peanut cultivation to mitigate viral diseases’ impact on yield and plant health. Continued monitoring and exploration of viral populations in peanuts are essential, particularly in the face of changing environmental conditions. This research forms a crucial foundation for future investigations into virus occurrence, diversity, and implications for effective disease management in peanut crops.

## Figures and Tables

**Table 1:** List of 15 peanut cultivars included in the virome study. All 15 distinct peanut cultivars were cultivated in the fields of the National Institute of Crop Science in Miryang, South Korea. Seed coat colors were also examined for each cultivar in the study.

Sample	Name of Cultivar	Seed Coat Color
P01	Daegwang	Normal
P02	Palgwang	Normal
P03	Agwang	Normal
P04	Backjung	Normal
P05	Pungan	Normal
P06	Sangan	Normal
P07	Sinpalkwang	Normal
P08	Keiol	Normal
P09	Daan	Normal
P10	Heuksaeng	Black
P11	Tamsil	Normal
P12	Sewon	Red
P13	Haeol	Normal
P14	Gowon	Normal
P15	Heukchan	Black

**Table 2:** Summary of raw data from the 15 libraries generated through RNA sequencing. The table provides the total read bases (in base pairs), total number of reads, GC content percentage, AT content percentage, Q20 percentage, and the respective Sequence Read Archive (SRA) accession numbers for each sample.

Sample	Total Read Bases (bp)	Total Reads	GC (%)	AT (%)	Q20 (%)	SRA acc. No.
P01	4,841,448,534	47,935,134	54.44	45.56	98.47	SRR26329875
P02	4,017,783,434	39,780,034	46.52	53.48	98.5	SRR26329874
P03	4,277,301,520	42,349,520	45.17	54.83	98.67	SRR26329868
P04	3,950,932,746	39,118,146	52.54	47.46	98.49	SRR26329867
P05	3,864,718,944	38,264,544	44.78	55.22	98.4	SRR26329866
P06	3,791,894,308	37,543,508	48.31	51.69	98.52	SRR26329865
P07	3,438,224,022	34,041,822	53.01	46.99	98.46	SRR26329864
P08	4,684,398,180	46,380,180	52.82	47.18	98.41	SRR26329863
P09	3,232,830,422	32,008,222	48.77	51.23	98.54	SRR26329862
P10	3,972,455,442	39,331,242	45.97	54.03	98.51	SRR26329861
P11	3,241,168,376	32,090,776	46.17	53.83	98.54	SRR26329873
P12	3,339,067,878	33,060,078	48.1	51.9	98.55	SRR26329872
P13	4,287,250,020	42,448,020	47.77	52.23	98.59	SRR26329871
P14	3,579,609,478	35,441,678	46.75	53.25	98.46	SRR26329870
P15	4,693,914,804	46,474,404	45.54	54.46	98.53	SRR26329869

**Table 3:** Virus-specific primer pairs used for RT-PCR detection of viruses infecting peanut plants in this study. For each primer pair, the reference genome accession number, primer name and sequence, genomic start and stop positions on the reference sequence, primer length, and expected amplicon size are indicated.

No.	Target Virus	Primer Name	Sequence of Primer	Start	Stop	Length	Target Size
1	BCMV NC_003397.1	BCMV_430_F	AATGGCACTTCACCGGATGT	9296	9315	20 mer	430 bp
		BCMV_430_R	CCATGCCAAGAAGTGTGTGC	9725	9706	20 mer	
2	CMV RNA3 NC_001440.1	CMV_626_F	AACCAGTGCTGGTCGTAACC	1274	1293	20 mer	626 bp
		CMV_626_R	CTCCAGATGTGGGAATGCGT	1899	1880	20 mer	
3	PeMoV NC_002600.1	PeMoV_471_F	TGGTTGGCGGACAGGTTATC	8788	8807	20 mer	471 bp
		PeMoV_471_R	GCGCTTTAGCTGATGTACGC	9258	9239	20 mer	
4	BrYV NC_016038.2	BrYV_405_F	AGCCTCTCGGACAACACAAC	3570	3589	20 mer	405 bp
		BrYV_405_R	TCATGCCATTCGATCCCGTT	3974	3955	20 mer	

## Data Availability

The raw datasets (SRR26329861 to SRR26329875) linked to the accession number PRJNA1026147 are accessible through the Sequence Read Archive (SRA) database at the National Center for Biotechnology Information (NCBI). The assembled viral genomes generated in this study have been submitted to GenBank under the accession numbers OR734859 to OR734903.

## Supplementary Materials

The following supporting information can be downloaded at: https://www.mdpi.com/article/10.3390/ijms27020890/s1.
