# Supplementary figures and images for: Exploring the Peanut Viromes Across 15 Cultivars in Korea
Source: Int J Mol Sci. 2026 Jan 15;27(2):890. doi: 10.3390/ijms27020890 (PMC12841197; doi:10.3390/ijms27020890)

# Figure S1

Group A  
1 isolate

Group B  
127 isolates

Group C  
32 isolates

# Figure S2

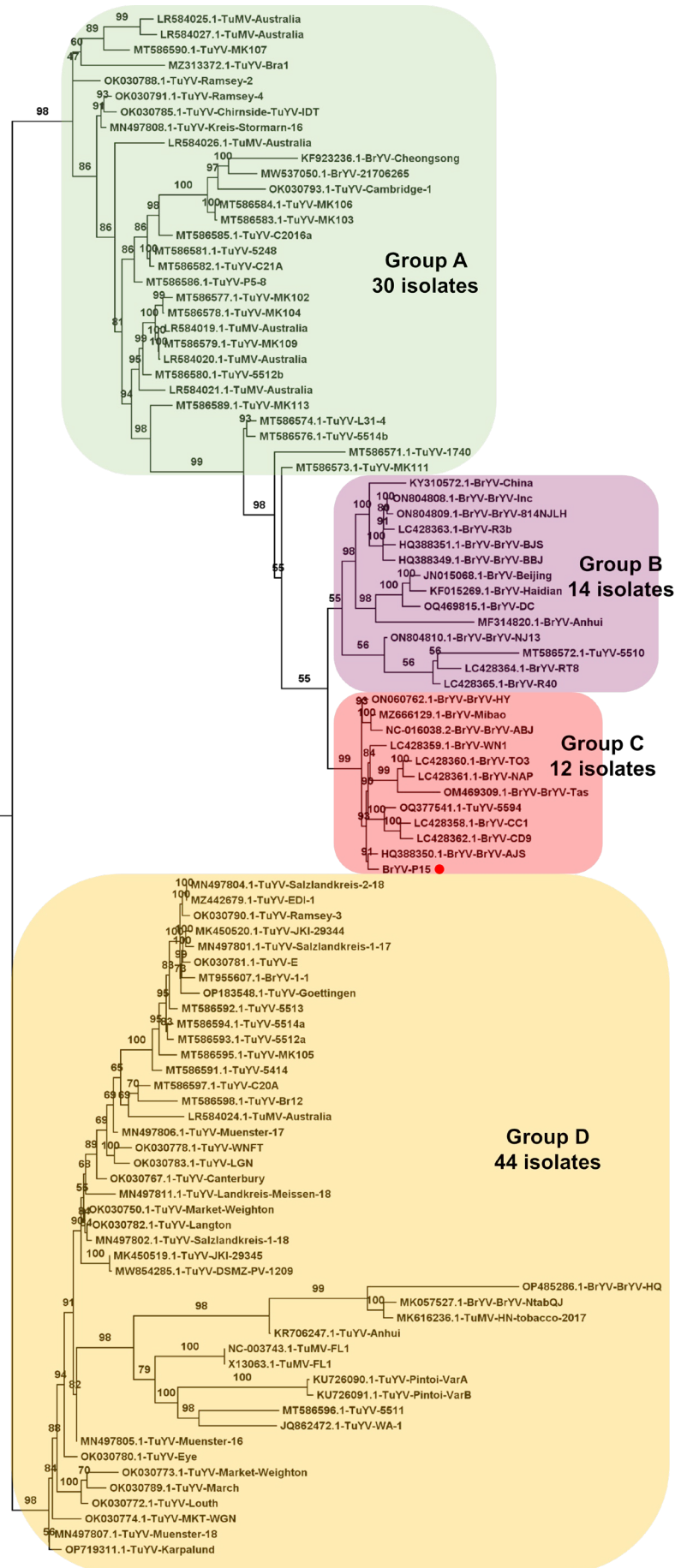

Figure S3

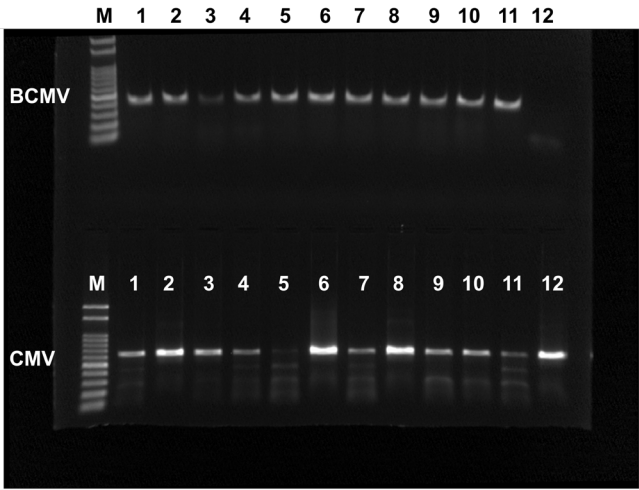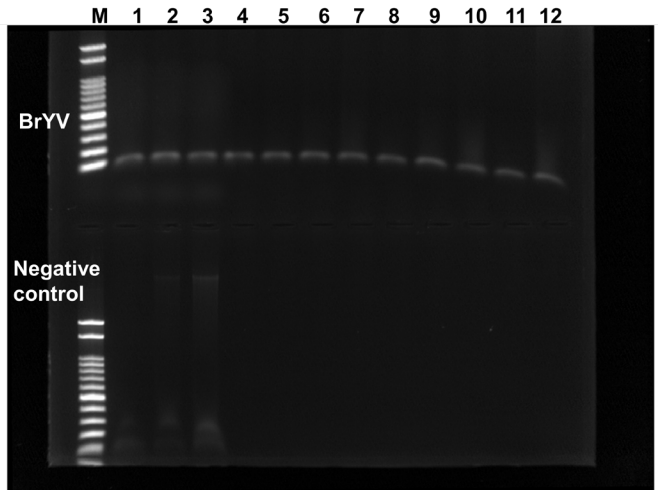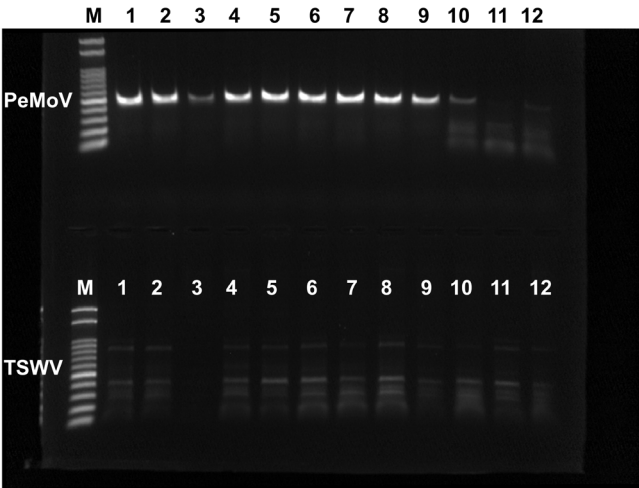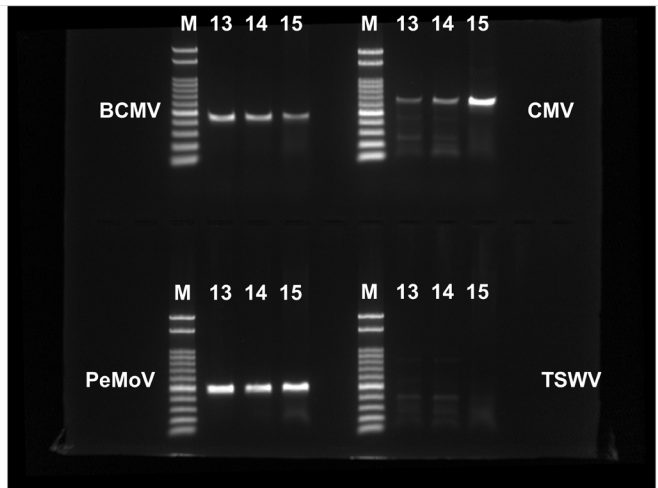

Supplement: Supplementary file 1 [file ijms-27-00890-s001.zip › Supplementary figures.pdf]
